# Changes in Minoxidil Prescribing After Media Attention About Oral Use for Hair Loss

**DOI:** 10.1001/jamanetworkopen.2023.12477

**Published:** 2023-05-09

**Authors:** Brianna M. Goodwin Cartwright, Michael Wang, Patricia Rodriguez, Sarah Stewart, Christopher M. Worsham, Nick Stucky, Anupam B. Jena

**Affiliations:** 1 Truveta, Inc, Bellevue, Washington; 2 Department of Health Care Policy, Harvard Medical School, Boston, Massachusetts; 3 Department of Medicine, Massachusetts General Hospital, Boston; 4 Division of Pulmonary and Critical Care Medicine, Massachusetts General Hospital, Boston; 5 National Bureau of Economic Research, Cambridge, Massachusetts

## Abstract

This cross-sectional study investigates rates of prescription of low-dose minoxidil after of publication of a newspaper article on this treatment.

## Introduction

Media coverage of health-related news may be associated with changes in clinician practices and patient behaviors.^1,2^ Evidence on the association of social media with these behaviors remains limited, however, despite increased interest in health-related news during the COVID-19 pandemic. On August 18, 2022, *The New York Times* published an article describing successful treatment experiences of several dermatologists and results of a small observational study of women with hair loss who received low-dose oral rather than topical minoxidil.^3,4,5^ We used prescription drug data to investigate changes in prescribing of oral minoxidil after the article, which was covered broadly by news and social media.^6^

## Methods

In this cross-sectional study, we identified adults prescribed oral minoxidil from January 1, 2021, to December 31, 2022, in the Truveta database, which holds electronic health records from member US health care systems. Included patients were treated in 8 health systems, primarily resided in 13 states, and received first-time oral minoxidil before (January to July 2022) or after (August to December 2022) publication of the article. Data were deidentified, and the Providence Institutional Review Board determined that this study was not human participants research. The study follows the STROBE reporting guideline for cross-sectional studies, including description of study design, setting, data sources, and statistical methods.

We calculated the weekly rate of first-time oral minoxidil prescriptions (No. prescriptions/No. outpatient encounters/week) for 2.5-mg and 5-mg tablets (10-mg tablets, used for hypertension, were excluded). We conducted interrupted time-series analyses of weekly prescription rates, accounting for autocorrelation using an autoregressive, integrated moving-average model. We used 2-sample *t* tests to test for a difference in means for 8 weeks before vs after article publication. *P* values reflected α = .025 in each tail. We made comparisons vs first-time low-dose finasteride hair loss medication (5-mg doses, which treat benign prostatic hyperplasia, were excluded) and antihypertensive medications (given that minoxidil is an antihypertensive).

## Results

Among 6541 patients with first-time oral minoxidil prescriptions (41.0% males; 36.8% aged 45-64 years; 7.5% Asian, 12.5% Black, and 65.2% White; 10.2% Hispanic), 2846 individuals received prescriptions in the 7 months before and 3695 individuals in the 5 months after publication, respectively (Table). The proportion of males (43.6% vs 37.7%) and White individuals (68.6% vs 60.8%) was higher after vs before publication. The proportion of individuals with comorbidities was lower after vs before publication (diabetes: 16.0% vs 22.1%; chronic kidney disease: 14.4% vs 22.3%; hypertension, 38.3% vs 46.7%).

**Table.** Characteristics of Study Population

Characteristic	Patients, No. (%)		
	Overall (N = 6541)	January to July 2022 (n = 2846)	August to December 2022 (n = 3695)
Sex			
Female	3840 (58.7)	1762 (61.9)	2078 (56.2)
Male	2685 (41.0)	1074 (37.7)	1611 (43.6)
Unknown	16 (0.2)	10 (0.4)	6 (0.2)
Age, y			
18-44	2107 (32.2)	926 (32.5)	1181 (32.0)
45-64	2404 (36.8)	1018 (35.8)	1386 (37.5)
≥65	2030 (31.0)	902 (31.7)	1128 (30.5)
Race			
Asian	492 (7.5)	192 (6.7)	300 (8.1)
Black or African American	816 (12.5)	469 (16.5)	347 (9.4)
White	4265 (65.2)	1730 (60.8)	2535 (68.6)
Other race^a^	475 (7.3)	241 (8.5)	234 (6.3)
Unknown	493 (7.5)	214 (7.5)	279 (7.6)
Ethnicity			
Hispanic or Latino	670 (10.2)	317 (11.1)	353 (9.6)
Not Hispanic or Latino	4848 (74.1)	2058 (72.3)	2790 (75.5)
Unknown	1023 (15.6)	471 (16.5)	552 (14.9)
Comorbidity			
Chronic kidney disease	1167 (17.8)	636 (22.3)	531 (14.4)
Chronic lung disease	458 (7.0)	196 (6.9)	262 (7.1)
Immunocompromised	263 (4.0)	121 (4.3)	142 (3.8)
Diabetes	1222 (18.7)	629 (22.1)	593 (16.0)
Hypertension	2743 (41.9)	1328 (46.7)	1415 (38.3)
Hyperlipidemia	2704 (41.3)	1150 (40.4)	1554 (42.1)
US census region			
Midwest	2059 (31.5)	971 (34.1)	1088 (29.4)
South	1207 (18.5)	542 (19.0)	665 (18.0)
West	2580 (39.4)	997 (35.0)	1583 (42.8)
Unknown or other	695 (10.6)	336 (11.8)	359 (9.7)

^a^ Other race includes American Indian or Alaska Native, Native Hawaiian or other Pacific Islander, and races that were classified as other by health systems.

The weekly rate of first-time minoxidil prescriptions per 10 000 outpatient encounters was significantly higher 8 weeks after vs 8 weeks before article publication overall (0.9 prescriptions [95% CI, 0.8-1.0 prescriptions] vs 0.5 prescriptions [95% CI, 0.4-0.6 prescriptions]; *P* < .001) and for males (1.1 prescriptions [95% CI, 0.9-1.3 prescriptions] vs 0.5 prescriptions [95% CI, 0.4-0.6 prescriptions], a 2.4-fold increase; *P* < .001) and females (0.8 prescriptions [95% CI, 0.7-0.9 prescriptions] vs 0.5 prescriptions [95% CI, 0.4-0.6 prescriptions], a 1.7-fold increase; *P* < .001) (Figure). After the initial increase associated with article publication, prescriptions decreased overall and for males and females. We did not observe similar increases in first-time finasteride or hypertension prescriptions.

**Figure.**
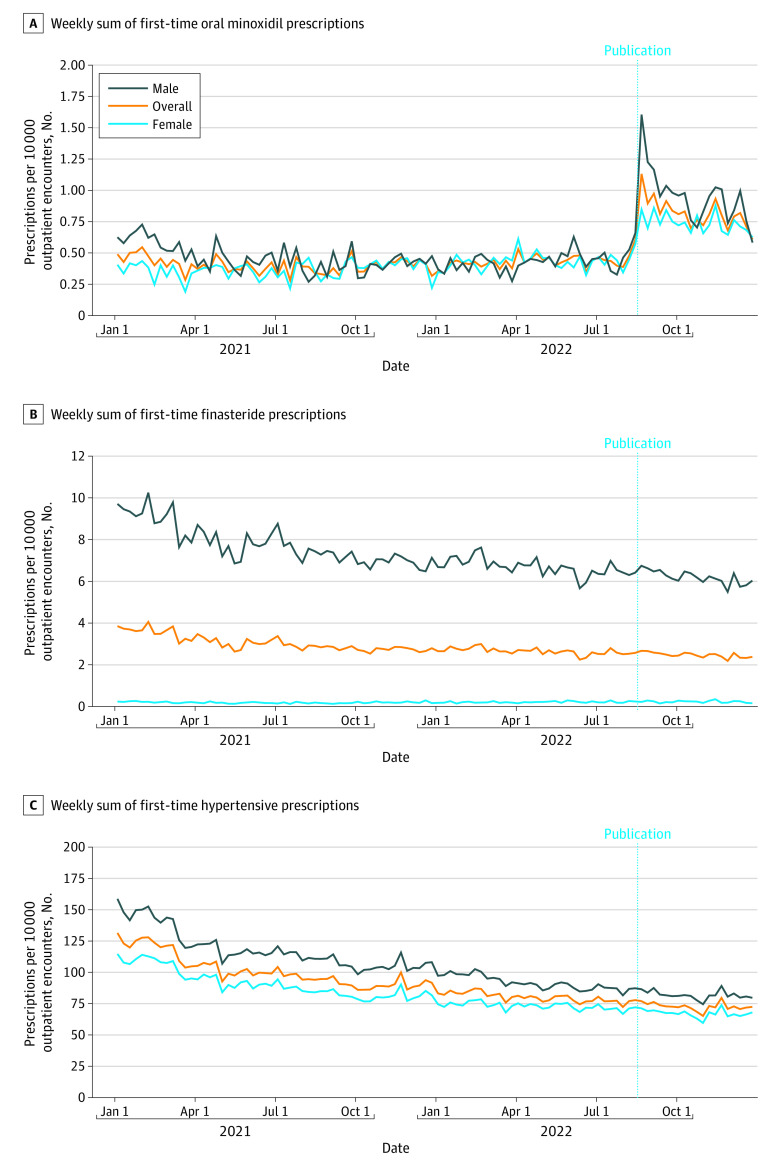
First-time Low-Dose Prescriptions

## Discussion

This cross-sectional study found that after a newspaper publication describing use of low-dose oral minoxidil to treat hair loss, there was an immediate increase in prescribing. Importantly, the article did not report new research findings or large-scale randomized evidence. Our findings suggest that media coverage alone, even without new research or with limited evidence, may be associated with immediate changes in prescribing, although they may not be sustained. Underlying factors associated with this change in prescription behavior from patients, doctors, or both and differences between males and females are important to understand. Socioeconomic factors, such as access to health care and education and income levels, may be associated with individuals seeking low-dose oral minoxidil after article publication. Study limitations include lack of generalizability to populations not studied, the possibility of an unobserved contemporaneous event associated with increased prescriptions of oral minoxidil, and the potential for misclassification of patients who divided 10-mg minoxidil tablets to treat hair loss.

## Notes

Supplement.
Data Sharing Statement
