# Supplementary material for: Changes in Minoxidil Prescribing After Media Attention About Oral Use for Hair Loss
Source: JAMA Netw Open. 2023 May 9;6(5):e2312477. doi: 10.1001/jamanetworkopen.2023.12477 (PMC10170338; doi:10.1001/jamanetworkopen.2023.12477)
Supplement: Supplement. — Data Sharing Statement [file jamanetwopen-e2312477-s001.pdf]

Data Sharing Statement

Goodwin Cartwright. Changes in Minoxidil Prescribing After Media Attention About Oral Use for Hair Loss. *JAMA Netw Open*. Published online May 9, 2023. doi:10.1001/jamanetworkopen.2023.12477

Data
Data available: No

© 2023 Cartwright BMG et al. *JAMA Network Open*.
